# The fungal literature-based occurrence database for southern West Siberia (Russia)

**DOI:** 10.3897/BDJ.9.e76789

**Published:** 2021-12-13

**Authors:** Nina Filippova, Dmitry Ageev, Sergey Bolshakov, Evgeny A. Davydov, Aleksandra Filippova, Ilya Filippov, Sergei Gashkov, Irina Gorbunova, Ludmila Kalinina, Nadezhda Kudashova, Ekaterina Palomozhnykh, Natalia Shabanova, Maria Tomoshevich, Olga Vayshlya, Anastasia Vlasenko, Vyacheslav Vlasenko, Irina Vorob'eva, Lidia Yakovchenko, Elena Zvyagina

**Affiliations:** 1 Yugra State University, Khanty-Mansiysk, Russia Yugra State University Khanty-Mansiysk Russia; 2 OOO (Limited Liability Company) "SIGNATEC", Novosibirsk, Russia OOO (Limited Liability Company) "SIGNATEC" Novosibirsk Russia; 3 Komarov Botanical Institute of the Russian Academy of Sciences, Saint Petersburg, Russia Komarov Botanical Institute of the Russian Academy of Sciences Saint Petersburg Russia; 4 Altai State University, Barnaul, Russia Altai State University Barnaul Russia; 5 Central Siberian Botanical Garden, Novosibirsk, Russia Central Siberian Botanical Garden Novosibirsk Russia; 6 Kemerovo State University, Kemerovo, Russia Kemerovo State University Kemerovo Russia; 7 National Research Tomsk State University, Tomsk, Russia National Research Tomsk State University Tomsk Russia; 8 Lomonosov Moscow State University, Moscow, Russia Lomonosov Moscow State University Moscow Russia

**Keywords:** occurrence, specimen, materialCitation, funga, fungi, Mycobiota, digitisation, biodiversity data mobilisation, GBIF

## Abstract

**Background:**

The paper presents the initiative on literature-based occurrence data mobilisation of fungi and fungi-related organisms (literature-based occurrences, Darwin Core MaterialCitation) to develop the Fungal literature-based occurrence database for the southern West Siberia (FuSWS). The initiative on mobilisation of literature-based occurrence data started in the northern part of West Siberia in 2016. The present project extends the initiative to the southern regions and includes ten administrative territories (Tyumen Region, Sverdlovsk Region, Chelyabinsk Region, Omsk Region, Kurgan Region, Tomsk Region, Novosibirsk Region, Kemerovo Region, Altai Territory and Republic of Altai). The area occupies the central to southern part of the West Siberian Plain and extends for about 1.5 K km from the west to the east from the eastern slopes of the Ural Mountains to Yenisey River and from north to south—about 1.3 K km. The total area equals about 1.4 million km^2^.

The initiative is actively growing in spatial, collaboration and data accumulation terms. The working group of about 30 mycologists from eight organisations dedicated to the data mobilisation was created as part of the Siberian Mycological Society (informal organisation since 2019). They have compiled the almost complete bibliographic list of mycology-related papers for the southern West Siberia, including over 900 publications for the last two centuries (the earliest dated 1800). All literature sources were digitised and an online library was created to integrate bibliography metadata and digitised papers using Zotero bibliography manager. The analysis of published sources showed that about two-thirds of works contain occurrences of fungi for the scope of mobilisation.

At the time of the paper submission, the database had been populated with a total of about 8 K records from 93 sources. The dataset is uploaded to GBIF, where it is available for online search of species occurrences and/or download. The project's page with the introduction, templates, bibliography list, video-presentations and written instructions is available (in Russian) at the web site of the Siberian Mycological Society. The initiative will be continued in the following years to extract the records from all published sources.

**New information:**

The paper presents the first project with the aim of literature-based occurrence data mobilisation of fungi and fungi-related organisms in the southern West Siberia. The full bibliography and a digital library of all regional mycological publications created for the first time includes about 900 published works. By the time of paper submission, nearly 8 K occurrence records were extracted from about 90 literature sources and integrated into the FuSWS database published in GBIF.

## Introduction

The mycological research in the southern part of West Siberia stems from isolated studies at the end of the 19^th^ century, yet regular and systematic research only began in the second half of the century. Over the following decades, several dozen researchers worked in the area and a total of over 1000 scientific works were published. The history of research of particular fungal groups was earlier described in a series of publications (Milovidova 1983, Davydov and Skachko 2014, Shirjaeva 2015, Sedel'nikova 2017). Below, we describe the history of mycological research in the southern part of West Siberia by traditionally-studied morphological or ecological groups.

### Overview of the mycological research reflected in the database

**The lichen diversity** in the region has been studied for more than hundred years. Irregular collections of lichens started at the end of the 19^th^ century by broad-scale collectors (from: Sedel'nikova 2017). This first period was summarised in a book chapter by Savich and Elenkin (1950). More systematic research of lichens started in the region in the second half of the 20^th^ century. In total, about 10 lichenologists worked in the area and published the results of the inventory or monitoring work. Systematic research of lichen diversity was made by Nellya V. Sedelnikova in several regions of southern West Siberia (occurrences summarised in Sedel'nikova 2017). Evgeny A. Davydov has been studying the lichen biota in Altai mountains (Davydov 2001, Davydov 2004, Davydov et al. 2007, Davydov and Printzen 2012, Davydov and Printzen 2012, Davydov 2012, Davydov et al. 2012) and Elena Y. Skachko in Altai plain (Barnaul vicinity) (Skachko 2003). Vera V. Koneva described the lichen communities and diversity in Tomsk Region (Koneva 2003). Eugene V. Barsukov studied lichen communities of pine forests in Novosibirsk Region (Barsukov 2001). In Omsk Region, lichen diversity of forest-steppe zone was studied by Natalia V. Sorokina (Sorokina 2001a, Sorokina 2001b). Ekaterina V. Romanova revealed bioindicator activity of lichens in Novosibirsk Region (Sedel'nikova and Romanova 2010). A detailed history of lichen research in Altai Territory provided in Davydov and Skachko (2014) and for West Siberia as a whole in (Sedel'nikova 2017). A number of important new records and species new for science were reported recently (Vondrák et al. 2016, Davydov and Konoreva 2017, Davydov and Yakovchenko 2017, Yakovchenko et al. 2017, Vondrák et al. 2019, Yakovchenko and Davydov 2018, Paukov et al. 2019, Yakovchenko et al. 2019, Paukov and Davydov 2020, Davydov et al. 2021).

**Agaricoid basidiomycetes** is a well, but unevenly, studied group in the region. Scientists performed targeted surveys on the group in Novosibirsk Region, Tomsk Region, Republic of Altai and Altai Territory. In the 1930s, the prominent mycologist of the 20^th^ century Rolf Singer visited the Altai Mountains, accompanied by Lubov' N. Vasiljeva. The collections made during the fieldwork were studied and cited in a number of papers, including the monumental "Das System der Agaricales III" (Singer 1943). In the 1960s, Nina V. Perova actually established the “mycological centre” in Novosibirsk which initiated the surveys of larger fungi of southern West Siberia, namely in Altai Republic, as well as Novosibirsk and Tomsk Regions with minor data from Kemerovo Region (Perova and Gorbunova 2001). In the 1990s, her successor Irina A. Gorbunova continued the work in various parts of the region, including several protected areas (Perova and Gorbunova 2007, Gorbunova et al. 2011, Gorbunova 2017, Gorbunova 2018). In the 2000s, Natalia P. Kutafieva started surveys in the Tomsk Region (Kosheleva and Kutaf'eva 2004). Later, Nadezhda N. Kudashova (Agafonova N.N.) with colleagues summarised all known data on larger fungi of the region (Kosheleva and Kutaf'eva 2004) and later added new information (Kudashova et al. 2016a, Kudashova et al. 2016b). In the borders of Kurgan, Omsk, Kemerovo and Tyumen Regions, only scattered data on agaricoid fungi can be found in several summarising works (Stepanova and Sirko 1977, Anonymous 1980, Mukhin 1993, Perova and Gorbunova 2001, Filippova et al. 2018). A detailed history of mycological studies in Sverdlovsk Region can be found in the summary by Olga S. Shiryaeva (Shirjaeva 2015).

**Gasteroid fungi** were studied by Yury A. Rebriev with co-authors (Rebriev and Gorbunova 2007, Shiryaev 2008a, Agafonova et al. 2011, Gorbunova and Rebriev 2018).

**Clavarioid fungi** were inventoried in different regions by Anton G. Shiryaev with colleagues (Shiryaev 2008b, Shiryaev and Gorbunova 2012, Vlasenko and Vlasenko 2017b).

The history of **aphyllophoroid basidiomycetes** research is described in Vlasenko (2013a). The southern West Siberia was less studied compared to the northern part, until recently. In the beginning of the 20^th^ century, the sporadic collection work was done by Nikolay N. Lavrov, Konstantin E. Murashkinskiy, M. K. Ziling, V. P. Dravert, V. V. Popov and others (from: Vlasenko 2013a). E. Zhukov (2002) and Zhukov (2005) were studying lignicolous basidiomycetes in Altai, Novosibirsk and Tomsk Regions in more detail at the end of the 20^th^ century. A relatively well-studied area by the school of aphyllophorologists is Sverdlovsk Region, importantly in mountains of the Urals (Mukhin 1993, Mukhin et al. 2003). In Altai Territory, the important inventories were made in the pine forests of forest-steppe zone (Vlasenko 2010, Vlasenko 2013a), in plantations and native forests of Novosibirsk (Vlasenko 2013b, Vlasenko 2014) and in different nature protected areas (Vlasenko and Vlasenko 2015, Vlasenko and Vlasenko 2017a, Vlasenko et al. 2019). In Tomsk Region, the first checklist of aphyllophoroid fungi was published in Agafonova et al. (2007b). **The biological activity** of agaricoid and aphyllophoroid basidiomycetes has been studied in Novosibirsk in different research projects, mentioning a few in the following references (Ibragimova et al. 2013, Troshkova et al. 2013, Protsenko et al. 2019).

Study of **fungal plant pathogens** in West Siberia started at the beginning of the 20^th^ century. Several regional checklists were created back then (Lavrov 1926, Murashkinskiy and Ziling 1928, Lavrov 1937, Lavrov 1938, Lavrov 1951a, Lavrov 1951b) and several monographs on special groups were published (Vasil'evskiy and Karakulin 1937, Transhel' 1939, Vasil'evskiy and Karakulin 1950). Further study of fungal plant pathogens in West Siberia resumed only in the 60-70s of the 20^th^ century. Plant pathogens in Tomsk City and Tomsk Region were studied by several authors (Milovidova and Melekhina 1971, Milovidova and Melekhina 1972, Sokolovskaya and Golovina 1973, Milovidova 1983). Fungal plant pathogens of woody plants in Novosibirsk were studied by Maria V. Nozdrenko (Nozdrenko 1960, Anonymous 1961, Nozdrenko 1964, Nozdrenko 1965, Anonymous 1974) and Ateo M. Zhukov (Zhukov 1978, Zhukov 1979). More recently, the diversity of fungal plant pathogens was advanced by Irina G. Vorobieva and Maria A. Tomoshevich (Tomoshevich and Vorob'eva 2005, Tomoshevich and Vorob'eva 2010, Tomoshevich and Banaev 2011, Vorob'eva et al. 2011, Tomoshevich 2012, Tomoshevich et al. 2013, Tomoshevich and Banaev 2013, Toropova et al. 2013, Tomoshevich and Banaev 2017, Vorob'eva and Tomoshevich 2017, Vorob'eva et al. 2018, Tomoshevich 2020, Tomoshevich and Vorob'eva 2020, Vorob'eva and Tomoshevich 2021, mentioning the most important works) and by Svetlana N. Nikitina, Elena Yu. Toropova (Nikitina 2008a, Nikitina 2008b, Toropova et al. 2013, Toropova et al. 2019, Toropova et al. 2021). A few publications were dedicated to the **soil communities of micromycetes** in Altai Territory, with one example given by Bilanenko and Georgieva (2005).

The history of **myxomycetes research** in West Siberia was presented in a paper (Vlasenko 2008). The first inventory of this group in Tomsk Region was made by Nikolay N. Lavrov (Lavrov 1927, Lavrov 1931). Recently, the systematic research of different ecological groups of myxomycetes in different regions was advanced by Anastasia V. Vlasenko with co-authors (Vlasenko 2011, Vlasenko and Novozhilov 2011, Vlasenko 2013, Vlasenko 2020, amongst others).

The description of the history of research was not intended to be complete and only describes the main fields of research of fungal diversity in the region and lists the key researchers and works. For a full mycological bibliography for the southern West Siberia, the reader is invited to read Suppl. material 1. This list is to be updated in the future and the latest version can be found in the working group' web page. For the history of mycological research in northern West Siberia, please refer to Filippova et al. (2020).

## Project description

### Title

The data mobilisation working group of the Siberian Mycological Society.

### Personnel

The working group of about 30 mycologists from eight organisations dedicated to the fungal literature-based records mobilisation initiative was created as part of the Siberian Mycological Society (informal organisation since 2019).

## Sampling methods

### Study extent

The project was aimed at mobilisation of species records accumulated in the course of previous mycological studies and published in peer-reviewed scientific literature from the beginning of research up to date (Filippova et al. 2021). The geography extended throughout the southern part of West Siberia, in the administrative borders of ten regions. About 900 publications were compiled in a bibliography and a digital library and the species occurrence records were extracted from about 90 selected works by the time of paper submission. The initiative will be continued in the following years to extract the records from all published sources.

### Step description

The following protocol was used to standardise and improve the mobilisation workflow:

1. The bibliography was compiled using Zotero bibliographic manager. Only published works (peer-reviewed papers, conference proceedings, PhD theses, monographs or book chapters) were selected. If possible, the sources were scanned and added to the library as PDF files.
2. The template of the FuSWS database was made with Google Sheets and simple Microsoft Excel templates. The Darwin Core standard was applied to the database field structure to accommodate the relevant information extracted from the publications. In total, 31 fields (see detailed description in Data resources) were selected to describe the literature-based occurrence data in the needed detail.
3. From the available publications related to the region, the only works with species occurrence reports were selected for the databasing purpose. The main source of occurrences were annotated species lists with exact localities of the records. However, different sorts of other species citations were also included, provided that they had the connection to any geography and could be georeferenced at least to the regional level.
4. Most of the occurrences were georeferenced, either from the coordinates provided in the paper or from the verbatim description of the field work locality. The georeferencing of the verbatim descriptions was made using Yandex or Google map services. Depending on the quality of georeference provided in publications, the coordinate uncertainty was estimated as follows: 1) the coordinate of a fruiting structure or a plot provided in the publication gives the uncertainty about 3-30 m; 2) the coordinate of the field work locality provided in publication gives the uncertainty between 500 m to 5 km; 3) the report of the species presence in a particular region gives the centroid of the area with the uncertainty radius to include its borders.
5. The locality names were reserved in the field «verbatimLocality» for accuracy.
6. When possible, the «eventDate» was extracted from the annotation data. Whenever this information was absent, the date of the publication was used instead, with the remarks in the «verbatimEventDate» field about the origin of the date.
7. The ecological features, habitat or relief were written in the «habitat» field and reserved in Russian.
8. The substrate is important feature of fungal occurrences and was extracted in the «fieldNotes» field.
9. Other annotation records, including the abundance, fruiting season and others, were accommodated in the «occurrenceRemarks» field.
10. The original scientific names reported in publications were filled in the «verbatimScientificName» field and reserved in the original database. This field was used to create the «ScientificName» field after spelling errors correction using the GBIF Species Matching tool. This tool was also used to create the additional fields of taxonomic hierarchy from species to kingdom, to fill in the «taxonRank» field and to synonymise according to the GBIF Backbone Taxonomy.
11. To track the digitisation process, a metadata worksheet was maintained. Each bibliographic record had a series of fields to describe the digitisation process and its results: the total number of extracted occurrence records, general description of the occurrence quality, presence of the observation date, details of georeferencing and the name of a person responsible for the digitisation.

## Geographic coverage

### Description

The dataset is limited by the administrative borders of ten regions (Tyumen Region, Sverdlovsk Region, Chelyabinsk Region, Kurgan Region, Omsk Region, Tomsk Region, Novosibirsk Region, Kemerovo Region, Altai Territory and Republic of Altai).

The region occupies the central to southern part of the West Siberian Plain. The area extends for about 1.5K km from the west to the east from the eastern slopes of the Ural Mountains to Yenisey River and from north to south – about 1.3K km. The total area equals about 1.4 m km^2^.

The area is very diverse in biogeographical terms, including several vegetation zones from steppe to taiga forest and mountain ecosystems. The relief in the central part is mainly a plain, but the south-eastern part of the area is occupied by several mountain systems of Altai, Salair, Kuznetsk Alatau and Gornaya Shoriya. The western part of West Siberia is bordered by the Ural Mountains.

**Figure 1.**
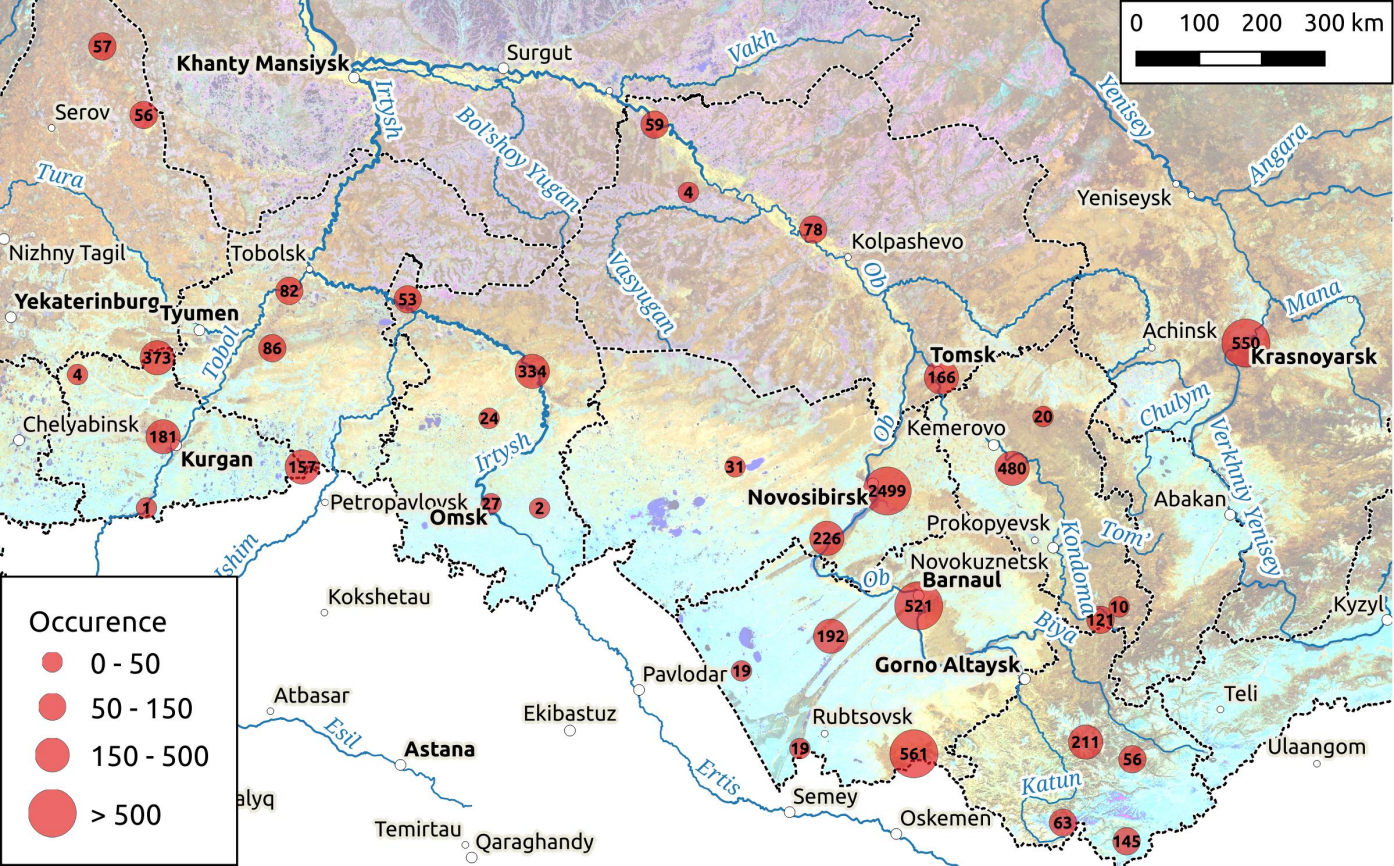
The distribution of the occurrence records from the FuSWS on Landsat satellite image of the area. The clustering of points was made within a radius of 100 km; the scale breaks were selected manually after plotting the frequency distribution histogram.

Most administrative divisions were covered by mycological research, but the intensity of the research varies (Fig. 1). Up to 85% of all records in the database currently made from five regions (Novosibirsk Region - 35%, Tomsk Region - 16%, Republic of Altai - 15%, Altai Territory - 11% and Kemerovo Region - 11% of total occurrences). Other regions are less covered in the database and the subject of future work.

### Coordinates

49.309 and 60.907 Latitude; 61.518 and 95.4909 Longitude.

## Taxonomic coverage

### Description

**Figure 2.**
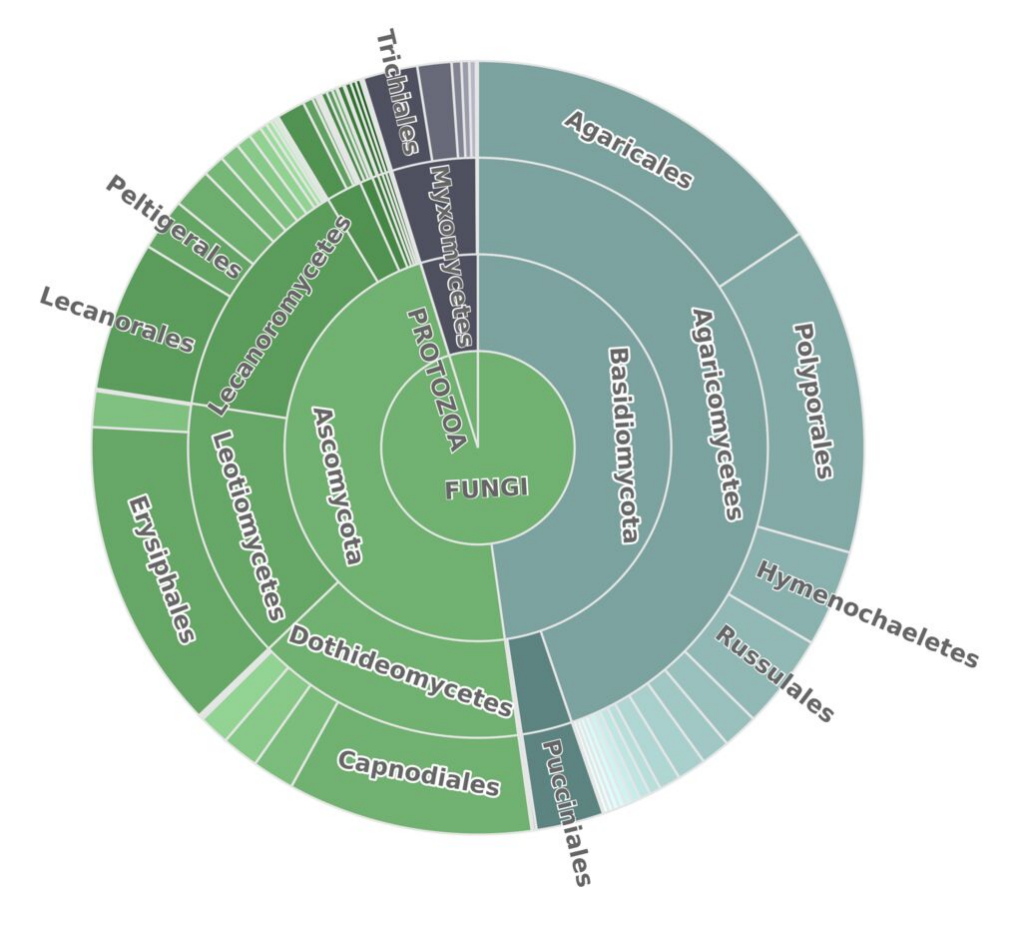
Taxonomic distribution of occurrences in the fungal literature-based occurrence database for the southern West Siberia.

According to the database summary report by the time of paper submission, there are occurrences of about 2200 species mobilised in the FuSWS database, which represent 800 genera, 230 families, 80 orders, 19 classes, five phyla and three kingdoms (Fungi, Protozoa, Chromista) (Fig. 2). The richest classes by number of occurrences are Agaricomycetes (44%), Dothideomycetes (15%), Leotiomycetes (15%) and Lecanoromycetes (14%). The richest ten families by number of occurrences are Erysiphaceae (13%), Polyporaceae (6%), Capnodiaceae (5%), Mycosphaerellaceae (5%), Meruliaceae (3%), Fomitopsidaceae (3%), Hymenochaetaceae (2%), Parmeliaceae (2%), Agaricaceae (2%) and Venturiaceae (2% of all occurrences).

## Temporal coverage

**Data range:** 1925-1-01 – 2019-1-01.

### Notes

About 90 publications for the last century.

## Usage licence

### Usage licence

Creative Commons Public Domain Waiver (CC-Zero)

### IP rights notes

This work is licensed under a Creative Commons Attribution (CC-BY) 4.0 License.

## Data resources

### Data package title

The Fungal Literature-based Occurrence Database for the southern West Siberia (Russia).

### Resource link

https://www.gbif.org/dataset/dd7d031f-b18f-4631-929a-049fcf00ac8f

### Alternative identifiers

http://ipt.ugrasu.ru:8080/ipt/resource?r=funsws

### Number of data sets

1

### Data set 1.

#### Data set name

The Fungal Literature-based Occurrence Database for the southern West Siberia (Russia).

#### Data format

Darwin Core

#### Number of columns

31

#### Description

The dataset includes a table in Darwin Core format with 31 original fields and about 8 K records.

**Data set 1.** 

Column label	Column description
occurrenceID	https://dwc.tdwg.org/terms/#dwc:occurrenceID; an identifier of a particular occurrence, unique within this dataset. We used simple 5-digit incremental number format.
basisOfRecord	https://dwc.tdwg.org/terms/#dwc:basisOfRecord; according to DwC recommendation, all literature-based records published to GBIF should have a value “MaterialCitation” (currently unavailable in IPT, but we will change it to this value in the future).
bibliographicCitation	https://dwc.tdwg.org/terms/#dcterms:bibliographicCitation; the bibliographic citation of a publication from which the occurrence was extracted, Elsevier - Harvard (with titles).
catalogNumber	https://dwc.tdwg.org/terms/#dwc:catalogNumber; the collection number or field number of the specimen, if provided in annotation (for example LE 255111 - specimen stored in Komarov Botanical Institude RAS).
coordinateUncertaintyInMetres	https://dwc.tdwg.org/terms/#dwc:coordinateUncertaintyInMeters; see "Sampling methods" for the description of the uncertainty calculation protocol.
countryCode	https://dwc.tdwg.org/terms/#dwc:countryCode; the standard code for the country in which the locality occurs (RU).
county	https://dwc.tdwg.org/terms/#dwc:county; the full, unabbreviated name of the next smaller administrative region than stateProvince (район).
decimalLatitude	https://dwc.tdwg.org/terms/#dwc:decimalLatitude; the geographic latitude provided in publication or determined from the provided geographic description; see "Sampling methods" for georeferencing details.
decimalLongitude	https://dwc.tdwg.org/terms/#dwc:verbatimLongitude; the geographic longitude provided in the publication or determined from the provided geographic description; see "Sampling methods" for georeferencing details.
eventDate	https://dwc.tdwg.org/terms/#dwc:eventDate; the full date of the observation event if provided in annotation or the year of publication itself, if absent in annotation of the record. In case the year of publication added, a corresponding remark was added in eventRemarks.
fieldNotes	https://dwc.tdwg.org/terms/#dwc:fieldNotes; the description of substrate.
geodeticDatum	https://dwc.tdwg.org/terms/#dwc:geodeticDatum; the geodetic datum upon which the geographic coordinates are given.
georeferencedBy	https://dwc.tdwg.org/terms/#dwc:georeferencedBy; a person who determined the georeference.
georeferenceProtocol	https://dwc.tdwg.org/terms/#dwc:georeferenceProtocol; see "Sampling methods" for georeferencing details.
georeferenceSources	https://dwc.tdwg.org/terms/#dwc:georeferenceSources; the resource used to georeference the locality.
habitat	https://dwc.tdwg.org/terms/#dwc:habitat; the description of habitat, including vegetation or relief.
identifiedBy	https://dwc.tdwg.org/terms/#dwc:identifiedBy; a person who identified the taxon.
kingdom	https://dwc.tdwg.org/terms/#dwc:kingdom; the full scientific name of the kingdom in which the taxon is classified.
locality	https://dwc.tdwg.org/terms/#dwc:locality; the original locality description of the collection place below county level, in English.
occurrenceRemarks	https://dwc.tdwg.org/terms/#dwc:occurrenceRemarks; other annotations to the record, including abundance, phenology etc.
recordedBy	https://dwc.tdwg.org/terms/#dwc:recordedBy; a person responsible for the original occurrence record, if present in annotation.
scientificName	https://dwc.tdwg.org/terms/#dwc:scientificName; the original names as provided in publication, but corrected for spelling mistakes using GBIF Species Matching tool.
stateProvince	https://dwc.tdwg.org/terms/#dwc:stateProvince; the name of the next smaller administrative region than country (область, край, республика).
taxonRank	https://dwc.tdwg.org/terms/#dwc:taxonRank; the taxonomic rank of the most specific name in the scientificName as it appears in the original publication.
verbatimElevation	https://dwc.tdwg.org/terms/#dwc:verbatimElevation; the original description of the elevation.
eventRemarks	https://dwc.tdwg.org/terms/#dwc:eventRemarks; information whether the eventDate was extracted from annotation or from a year of publication.
verbatimLocality	https://dwc.tdwg.org/terms/#dwc:verbatimLocality; the original locality description of the collection place below county level, reserved in original language.
verbatimLatitude	https://dwc.tdwg.org/terms/#dwc:verbatimLatitude; the original latitude format as it was provided in a publication.
verbatimLongitude	https://dwc.tdwg.org/terms/#dwc:verbatimLongitude; the original longitude format as it was provided in a publication.
verbatimEventDate	https://dwc.tdwg.org/terms/#dwc:verbatimEventDate; the original representation of the date as it was provided in a publication.
language	https://dwc.tdwg.org/terms/#dcterms:language; a language of the dataset, which is Russian for some of the fields (bibliographicReference, verbatimLocality, occurrenceRemarks, habitat) and English for other fields.

## Supplementary Material

89E0ACC6-B10A-5909-9220-9CC8D9EE807D 10.3897/BDJ.9.e76789.suppl1 Supplementary material 1 The bibliography of mycological research in southern West Siberia Data type Bibliography Brief description The bibliography presents all mycological scientific publications (journal papers, conference proceedings, PhD theses, monographs and book chapters) related to the mycological research in southern West Siberia from the beginning of research to date. File: oo_620525.txt https://binary.pensoft.net/file/620525 Nina Filippova, Dmitry Ageev, Sergey Bolshakov, Evgeny A. Davydov, Aleksandra Filippova, Ilya Filippov, Sergei Gashkov, Irina Gorbunova, Ludmila Kalinina, Nadezhda Kudashova, Ekaterina Palomozhnykh, Natalia Shabanova, Maria Tomoshevich, Olga Vayshlya, Anastasia Vlasenko, Vyacheslav Vlasenko, Irina Vorob'eva, Lidia Yakovchenko, Elena Zvyagina
